# A Manual of Otology

**Published:** 1902-11

**Authors:** 


A MANUAL OF OTOLOGY. By GORHAM BACON, A. M., M. D, Professor of Otology in Cornell University Medical College, New York. With an introductory Chapter by Clarence J. Blake, M. D., Professor of Otology in Harvard Medical School, Boston. New (3d) edition. In one 12mo volume of 437 pages, with 120 engravings and seven plates in colors and monochrome. Philadelphia and New York: Lea Brothers & Co. 1902. [Price, cloth, $2.25 net.]

The third edition of Bacon's Manual leaves little to be desired in such a work. Its arrangement is ideal, the illustrations well chosen and the text unusually clear and concise, the subjects being handled with a practical straight forwardness that is refreshing. The chapter on intracranial complications is a very important feature and one that the student or busy practitioner will find of great value; the idea of including the surrounding parts likely to be affected, is an extremely good one and worthy of emulation. The object of "affording a work of easy reference in lieu of the more exhaustive treatises," is certainly attained; for, with the terseness and directness of a quiz compend, is given the subject-matter of a treatise. L. B., Jr.

